# Coronaviruses and gastrointestinal diseases

**DOI:** 10.1186/s40779-020-00279-z

**Published:** 2020-10-14

**Authors:** Xi Luo, Guan-Zhou Zhou, Yan Zhang, Li-Hua Peng, Li-Ping Zou, Yun-Sheng Yang

**Affiliations:** 1 Department of Gastroenterology and Hepatology, the First Medical Center, Chinese PLA General Hospital, Beijing, 100853 China; 2 Department of Pediatrics, the First Medical Center, Chinese PLA General Hospital, Beijing, 100853 China

**Keywords:** Mammal coronaviruses, Human coronaviruses, Digestive system, Coronavirus disease 2019 (COVID-19)

## Abstract

The effects of coronaviruses on the respiratory system are of great concern, but their effects on the digestive system receive much less attention. Coronaviruses that infect mammals have shown gastrointestinal pathogenicity and caused symptoms such as diarrhea and vomiting. Available data have shown that human coronaviruses, including the newly emerged SARS-CoV-2, mainly infect the respiratory system and cause symptoms such as cough and fever, while they may generate gastrointestinal symptoms. However, there is little about the relation between coronavirus and digestive system. This review specifically addresses the effects of mammalian and human coronaviruses, including SARS-CoV-2, on the digestive tract, helping to cope with the new virus infection-induced disease, COVID-19.

## Background

The coronavirus disease 2019 (COVID-19) pandemic caused by severe acute respiratory syndrome coronavirus 2 (SARS-CoV-2) has resulted in severe adverse impacts worldwide. Available data have shown that patients with COVID-2019 present respiratory symptoms and fever; however, approximately 8.8–60.6% of patients suffer from gastrointestinal symptoms, such as decreased appetite, nausea, vomiting, and diarrhea, as well as possible abnormal liver function [1–3]. Although the impacts of SARS-CoV-2 on the gastrointestinal (GI) tract remain unclear, studies of other coronaviruses have revealed a close relation to GI symptoms. We reviewed the coronaviruses that cause GI disorders in mammals or humans to facilitate coping with SARS-CoV-2 infection.

Coronaviruses (CoV) are enveloped, single-stranded, positive-sense RNA viruses that belong to the order Nidovirales. They contain the most abundant RNA genome, with lengths from 27 to 32 kb among all known viruses, and are members of the family Coronaviridae, subfamily Coronavirinae. This subfamily consists of four genera, *Alphacoronavirus*, *Betacoronavirus*, *Gammacoronavirus* and *Deltacoronavirus* (α-, β-, γ- and δ-CoV). Specifically, α- and β- coronaviruses mainly infect the human respiratory system, mammalian intestine, and central nervous system; γ- and δ- coronaviruses primarily infect birds (or poultry). The coronaviruses infecting humans can cause severe diseases. To date, seven coronaviruses (HCoVs) have been reported in humans, namely, human coronavirus 229E (HCoV-229E), human coronavirus NL63 (HCoV-NL63), human coronavirus HKU1 (HCoV-HKU1), human coronavirus OC43 (HCoV-OC43), Middle East respiratory syndrome coronavirus (MERS-CoV), severe acute respiratory syndrome coronavirus (SARS-CoV), and the recently identified SARS-CoV-2 [4, 5].

## Enteric coronavirus in mammals

Porcine coronaviruses, such as porcine transmissible gastroenteritis virus (TGEV), porcine epidemic diarrhea virus (PEDV), and swine acute diarrhea syndrome coronavirus (SADS-CoV), are classified as genus *Alphacoronavirus*, while porcine deltacoronavirus (PDCoV) belongs to genus *Deltacoronavirus*. These porcine coronaviruses can infect piglets of all ages by invading the GI tract, with clinical signs of diarrhea, vomiting, and anorexia, resulting in dehydration, metabolic acidosis, and hyperkalemia and eventually leading to death. Scattered intestinal epithelial cell necrosis and villus damage are the primary pathological manifestations of porcine coronavirus infection [6–8]. There is a higher incidence of porcine coronavirus infection during winter and spring; piglets are particularly more susceptible than adult pigs to porcine coronavirus, with a higher mortality rate [6]. In addition, mixed infection of these four porcine coronaviruses is common among infected pig herds. Current evidence indicates that porcine coronaviruses can be transmitted through the fecal-oral route and infect small intestinal epithelial cells due to resistance to low pH and proteolytic enzymes, causing osmotic diarrhea in infected pigs and even death [9]. In addition to fecal-oral transmission, PDCoV can also be transmitted through the respiratory tract and induce pneumonia [10]. Etiological treatment with additional nutrient supply should be followed to lower mortality, which is caused by secondary infection and dehydration due to diarrhea. It is essential to maintain water and electrolyte balance [9]. In addition, probiotics, such as Lactobacillus, are reported to be effective in treating porcine coronavirus diarrhea [11].

Canine coronaviruses (CCoV) involve two serotypes, CCoV-I and CCoV-II, which belong to α-coronavirus. CCoV-II can be further divided into CCoV-IIa (the classical biotypes and pantropic biotypes) and CCoV-IIb. Classical CCoV-IIa is restricted to the small intestine, causing symptoms such as decreased appetite, vomiting, watery diarrhea, and dehydration. Pantropic CCoV-IIa causes subacute bronchial pneumonia and leukopenia (mainly CD4^+^ T cells), as well as hemorrhage and necrosis in multiple organs (e.g., kidney and liver) [12, 13]. Canine coronavirus disease generally has a high incidence rate, has a low mortality rate, and presents mild gastrointestinal symptoms. Severe cases are often accompanied by coinfection with other viruses. Prevention and treatment of canine coronavirus disease include strengthening hygiene, quarantine, and maintaining water and electrolyte balance. Specific antiviral therapies, such as vaccines and monoclonal antibodies, are also used [12].

Feline coronavirus (FCoV) includes feline enteric coronavirus (FECV) and feline infectious peritonitis virus (FIPV), both of which are α-coronaviruses. FECV usually causes mild intestinal symptoms in cats but self-limiting recovery due to weak toxicity. In contrast, the toxicity of FIPV is generally strong and leads to a high fatality rate, causing systemic inflammatory diseases are known as feline infectious peritonitis (FIP). Generally, FCoV replicates in host intestinal epithelial cells and is excreted with feces [14, 15]. At the early stage of FIP, hosts present mild symptoms of GI infection. Later, hydrothorax and ascites will appear, mesenteric lymph nodes will be enlarged, and multiple organs in the body will be involved [16]. It is worth mentioning that some cats present oral lesions, such as gingivitis and mucosal ulcers, and halitosis as the most obvious symptoms, which might be strong evidence for the existence of virus infection [17]. The recommended treatment includes antiviral agents, secondary infection prevention, osmotic pressure increase, and fluid infusion. In addition, glucocorticoids are the main immunosuppressive agents in the treatment of FIP [16].

Bovine coronavirus (BCoV) is a member of the genus *β-coronavirus,* which has high similarity with human coronavirus OC43 in antigenicity and gene sequence [18]. BCoV can be transmitted through the respiratory and digestive tracts and damages small intestinal epithelial cells or respiratory epithelial cells, causing diarrhea, intestinal bleeding, fever, and sometimes cough and runny nose. BCoV RNA could be detected in the stool of both healthy and diarrheal cattle, and the detection rate was higher in stool samples from diarrheal cattle [19]. Currently, there is no specific treatment, and symptomatic relief is the primary therapy. In addition, liquid supplementation is essential in severe conditions. Some studies have suggested that *Lactobacillus* preparations are effective in treating bovine diarrhea, but this assertion is still controversial [20].

## Coronavirus in the human gastrointestinal tract

The *α-coronaviruses* HCoV-229E and HCoV-NL63 were isolated in 1965 and 2004, respectively [21, 22]. Epidemiological investigation shows that humans are generally susceptible to these two viruses, and nearly all children have been infected with HCoV-229E and HCoV-NL63 at an early stage [23]. HCoV-229E mainly causes mild respiratory symptoms, including headache, runny nose, sore throat, fever, and cough [24, 25]. HCoV-NL63 infects the lower respiratory tract and causes bronchiolitis, pneumonia, etc. [26].

Both HCoV-OC43 and HCoV-HKU1 belong to lineage A of the genus *β-coronavirus*, which were found in 1967 and 2005, respectively [27, 28]. Although the source of HCoV-OC43 remains uncertain, studies have suggested that HCoV-OC43 is closely related to lineage A of the genus *β-coronavirus*, especially bovine coronavirus (BCoV) [18]. HCoV-OC43 typically causes mild respiratory symptoms but infects and leads to severe lower respiratory tract infection in some immunocompromised cases [29]. In addition to upper respiratory tract infection, HCoV-HKU1 can also causes lower respiratory tract infections, such as pneumonia and bronchitis [29], with occasional reported fatal cases. It is rarely reported that the above four human coronaviruses cause digestive disorders [30]. However, approximately 25–38% of patients infected with these four human coronaviruses develop gastrointestinal symptoms [31].

## SARS-CoV

SARS-CoV was isolated and identified in 2003, belonging to lineage B of the genus *β-coronavirus*. Fever and respiratory symptoms are the most common signs of the infection, and additional GI symptoms also appear as anorexia, nausea, vomiting, abdominal pain, and diarrhea. A retrospective study investigated 752 people with SARS-CoV infection and indicated that approximately 20% of patients had diarrhea in their initial stages [32]. Another investigation showed that 55 of 75 patients with SARS-CoV infection presented watery stools approximately 1 week after onset [33]. Altogether, approximately 20–25% of SARS patients will have diarrhea [2].

The majority of SARS-CoV enters human cells through angiotensin-converting enzyme 2 (ACE2), which is expressed on lung and gastrointestinal cells [34]. Currently, reverse transcription-polymerase chain reaction (RT-PCR) tests are the primary methods of pathogenic detection by using upper respiratory tract specimens via throat swabs, lower respiratory tract specimens such as bronchoalveolar lavage fluid, and blood, urine, or fecal specimens. SARS-CoV is persistently positive with intestinal specimen tests for the longest duration, from day 5 after the onset of illness to over 30 days, with the peak at day 11 in some patients [35]. In an investigation of 67 patients who were diagnosed with SARS through stool RNA tests, 65 patients (97%) were positive at day 14 after the onset of illness [35]. In another cohort study of 75 patients with SARS, nasopharyngeal aspirates from 51 patients showed positive results on day 14 with a nucleic acid test. Subsequently, 15 patients were followed up to day 21, and stool samples from 10 of them (67%) remained positive [33]. This result suggests that the release of the virus via the fecal system occurs for a longer period of time than that by the airways. As a result, detection from different sample types is necessary, and the timeliness of detection is not the same. In addition, patients with gastrointestinal symptoms should have a longer isolation period. Comprehensive judgment is needed during practice.

## MERS-CoV

MERS-CoV was first detected in Saudi Arabia in September 2012, which belongs to lineage C of *Betacoronavirus* and binds to the dipeptidyl peptidase 4 (DPP4) receptor [36]. Based on existing evidence, patients with MERS-CoV infection present with fever; respiratory symptoms, including cough, sputum, and dyspnea; and gastrointestinal symptoms, such as anorexia, nausea, vomiting, abdominal pain, and diarrhea, and general shock or kidney damage may also appear during the illness [36]. In a retrospective investigation of 12 patients with severe SARS, 11 patients had shock symptoms, and 7 had acute kidney injury [36]. Another study reported the symptoms of 47 patients with MERS: 12 (26%) had diarrhea, 10 (21%) had vomiting, and 8 (17%) had abdominal pain [37]. Approximately 25% of patients with MERS have developed diarrhea or abdominal pain at the time of consultation. Some patients initially present with fever and gastrointestinal symptoms, followed by more severe respiratory symptoms [37]. Based on RNA tests, approximately 14.6% of stool samples from patients with MERS showed positive results [38].

## SARS-CoV-2

SARS-CoV-2 belonging to *Betacoronavirus* was first reported in 2019 in China, which has significant genetic characteristics distinguished from those of SARS-CoV and MERS-CoV. The genome sequence of SARS-CoV-2 shares over 85% homology with that of bat SARS-CoV-like viruses (bat-SL-CoVZC45) [39]. The infectious disease (COVID-19) caused by SARS-CoV-2 has similar clinical signs as SARS-CoV infection, including fever and respiratory symptoms, such as cough, sputum, shortness of breath, and breathing difficulties. Furthermore, COVID-19 patients present various digestive symptoms. However, reports on the incidence of gastrointestinal symptoms in COVID-19 patients vary widely. COVID-19-associated diarrhea occurrence has been reported at 3.0% [40], 7.3% [1], 10.1% [1], 2.0% [41], and 49.5% [42] in different studies. A study from a single center in Wuhan on 305 patients reported approximately 22.2% [42]. In a large sample study of 1099 patients across China, the incidence of gastrointestinal symptoms was 3.8% for patients with diarrhea symptoms [2]. This discrepancy may be attributed to the different sample amounts of each study and the awareness of some mild gastrointestinal symptoms. In some cases, patients even presented with gastrointestinal symptoms alone at the early stage of onset, including anorexia, nausea, vomiting, and diarrhea [41]. Thus, we should pay additional attention to mild GI symptom patients, and COVID-19 screening is strongly recommended for patients with GI symptoms in the epidemic region. The related gastrointestinal symptoms of SARS-CoV-2 may be associated with its receptor. The binding receptor in the human body for SARS-CoV-2 is ACE2, which is highly expressed in type II alveolar epithelial cells and esophageal epithelial cells, and high expression was also found in the ileum and colon [43]. The positive results of gastrointestinal tract samples provide evidence for enteric transmission. Elsewhere, the RNA test for the stool sample of the first COVID-19 patient in the United States showed positive results on day 7 after onset, suggesting the possibility of SARS-CoV-2 transmission in the digestive tract [44]. A similar possibility was found in a study of sixty-two COVID-19 patients in China. There were two mild COVID-19 patients and two severe COVID-19 patients (6.5%) that showed positive results of the fecal RNA test, and only one of the four patients had diarrhea symptoms [2]. In another report, anal swabs from four patients were positive for RNA tests, of which two patients were positive for RNA tests for the esophagus, stomach, duodenum, and rectal mucosa samples [2]. These results indicate that SARS-CoV-2 has potential gastrointestinal transmission. In terms of pathological analysis, one autopsy report of a COVID-19 patient showed segmental dilatation and stenosis of the small intestine [45]. Although autopsy and puncture tissue pathological samples are limited, pathological observations have shown that the mucosal epithelium of the esophagus, stomach, and intestine are degenerated, necrotic, and shed [45]. These studies suggest the possibility of SARS-CoV-2 directly affecting the intestinal mucosa and causing intestinal damage. Similar to the case for SARS-CoV, the detection results of different body part samples are not the same. It was reported that three patients with SARS-CoV-2 had a negative nucleic acid test on throat swab samples after treatment. However, their fecal nucleic acid test results were still positive [46], suggesting that in specimens from different sites, the nucleic acid conversion time is not synchronized and that the fecal nucleic acid positive duration may be longer. It tells us that there are some characteristics deserving attention in the diagnosis and quarantine of COVID-19 patients with gastrointestinal symptoms.

## Conclusions

This review describes eight coronaviruses that can infect mammals and cause gastrointestinal symptoms and seven human coronaviruses (see Table 1 for details). Coronaviruses that infect mammals have definite intestinal pathogenicity and can cause related clinical manifestations, such as vomiting and diarrhea, in animals, with high lethality. In addition, some coronaviruses in mammals can be transmitted through digestive and respiratory contaminants. The treatments of mammalian disease mainly comprise symptomatic therapy, fluid supplementation and internal environment maintenance, and probiotics are also useful. Gastrointestinal symptoms, receptor binding, positive nucleic acid results and pathological evidence strongly reveal the strong relationship between SARS-CoV-2 and the gastrointestinal tract. The pathogenesis of the digestive disorders induced by coronaviruses needs to be investigated and revealed.

**Table 1:** Comparison of human and mammalian coronaviruses

Name	Taxonomy	Host	Gastrointestinal symptoms	Other system symptoms	Ref.
HCoV-229E	α	Human/bat	Rarely mild nausea, vomiting, diarrhea	Common cold symptoms, such as headache, tiredness, rhinorrhea, sore throat, fever and cough	[21, 24, 25]
HCoV-OC43	*β-coronavirus* lineage A	Human/rat, cow	Rarely mild nausea, vomiting, diarrhea	Common cold symptoms, such as headache, tiredness, rhinorrhea, sore throat, fever and cough	[27, 29]
SARS-CoV	*β-coronavirus* lineage B	Human/bat, masked civet	Abdominal pain, nausea, vomiting, diarrhea	Fever, muscle weakness, tiredness, headache, chill, dyspnea, pneumonia, multiple organ dysfunction	[32–35]
HCoV-NL63	α	Human/bat	Rarely mild nausea, vomiting, diarrhea	Common cold symptoms, such as headache, tiredness, rhinorrhea, sore throat, fever and cough	[22, 26]
HCoV-HKU1	*β-coronavirus* lineage A	Human	Rarely mild nausea, vomiting, diarrhea	Common cold symptoms, such as headache, tiredness, rhinorrhea, sore throat, fever and cough	[28]
MERS-CoV	*β-coronavirus* lineage C	Human/bat, camel	Abdominal pain, nausea, vomiting, diarrhea	Fever, muscle weakness, tiredness, headache, chill, dyspnea, pneumonia, multiple organ dysfunction	[36–38]
SARS-CoV-2	*β-coronavirus* lineage B	Human	Abdominal pain, nausea, vomiting, diarrhea	Fever, muscle weakness, tiredness, headache, chill, dyspnea, pneumonia, multiple organ dysfunction	[1–3, 39–45]
PEDV	α	Porcine	Diarrhea, vomiting	–	[6–8]
TGEV	α	Porcine	Diarrhea, vomiting	–	[6–8]
SADS-CoV	α	Porcine/bat	Acute severe diarrhea, vomiting	Tiredness, rapid weight loss	[6–8]
PDCoV	δ	Porcine	Diarrhea, vomiting	–	[9–11]
FIPV	α	Feline	Diarrhea, vomiting	Anorexia, weight loss, dehydration, consecutive fever in the early stage; pleural effusion, ascites, enlarged mesenteric lymph nodes, multiple organ dysfunction, such as in the eyes, kidneys, liver or central nervous system, in the later stage	[15, 16]
FECV	α	Feline	Mild intestinal infection, diarrhea, vomiting	–	[15, 16]
CCoV	α	Canine	Diarrhea, sometimes vomiting	–	[12, 13]
BCoV	β	Bovine	Diarrhea, sometimes hematochezia	Fever, milk production reduction, anorexia, sometimes cough and rhinorrhea	[18–20]

SARS-CoV. Severe acute respiratory syndrome coronavirus; MERS-CoV. Middle East respiratory syndrome coronavirus; PEDV. Porcine epidemic diarrhea virus; TGEV. Transmissible gastroenteritis virus; SADS-CoV. Swine acute diarrhea syndrome coronavirus; PDCoV. Porcine delta coronavirus; FIPV. Feline infectious peritonitis virus; FECV. Feline enteric coronavirus; CCoV. Canine coronavirus; BCoV. Bovine coronavirus

## Data Availability

The data and materials used during the current review are all available in this review.

## Abbreviations

ACE2: Angiotensin-converting enzyme 2
BCoV: Bovine coronavirus
CCoV: Canine coronavirus
CoV: Coronaviruses
COVID-19: Coronavirus disease 2019
DPP4: Dipeptidyl peptidase 4
FECV: Feline enteric coronavirus
FIP: Feline infectious peritonitis
FIPV: Feline infectious peritonitis virus
GI: Gastrointestinal
HCoV: Human coronavirus
HCoV-229E: Human coronavirus 229E
HCoV-HKU1: Human coronavirus HKU1
HCoV-NL63: Human coronavirus NL63
HCoV-OC43: Human coronavirus OC43
MERS-CoV: Middle East respiratory syndrome
PDCoV: Porcine delta coronavirus
PEDV: Porcine epidemic diarrhea virus
SADS-CoV: Swine acute diarrhea syndrome coronavirus
SARS-CoV: Severe acute respiratory syndrome coronavirus
SARS-CoV-2: Severe acute respiratory syndrome coronavirus 2
TGEV: Transmissible gastroenteritis virus
